# Social determinants of mental health: a systematic review about current main predictors and different health contexts on the world

**DOI:** 10.1192/j.eurpsy.2026.10462

**Published:** 2026-07-31

**Authors:** M. C. B. Maia, B. E. Bandeira, L. A. Brito, M. Coufalíková

**Affiliations:** 1 Universidade de Brasilia, Brasilia, Brazil; 2 faculty of medicine masaryk university, Brno, Czech Republic

## Abstract

**Introduction:**

Good mental health is an essential part of overall health and well-being. It is shaped by social, economic, and physical environments that operate at different stages of life, as shown in image 1 (Kirkbride JB et al. World Psychiatry 2024; 23:58–90). Risk factors for many common mental health conditions are closely tied to social inequalities – the greater the inequality, the greater the risk. This report reviews the main social determinants of health, defined as “the circumstances in which people are born, grow, work, live and age, and their access to power, money and resources”.

**Objectives:**

The aim is to map the social factors most closely linked to the onset and exacerbation of mental health conditions. The project will also analyse how these factors relate to socioeconomic inequalities, gender, ethnicity, and other social markers in different contexts around the world.

**Methods:**

This systematic literature review was conducted by searching the PubMed/MEDLINE, Scopus, Web of Science and PsycINFO databases for articles published since 2017. The keywords used were ‘social determinants of health’, ‘mental health’ and ‘predictors’.

**Results:**

Poverty, socioeconomic inequality, lack of education, and food insecurity are consistently identified as key predictors of mental disorders, including depression and anxiety (Lund et al. Lancet Psychiatry 2018; 5 357–369). Exposure to multiple forms of social disadvantage is cumulative and exacerbates negative mental health outcomes, reflecting a pattern of “social causality” where living conditions influence health. Additionally, distinct geographic and cultural contexts modulate the manifestation and prevalence of mental disorders (Kirkbride et al. World Psychiatry 2024; 23 58–90). The application of the Social Ecological Model offers a useful lens for understanding how HSDs operate at multiple levels—individual, community, institutional, and social—to shape the mental health of adolescents and other age groups, according to table 1 (Robins et al. Journal of Human Services 2024). School environment, family relationships, and community integration play important roles as protective or risk factors for the development of psychological distress between children and teenagers (Robins et al., 2024).

**Image 1:**
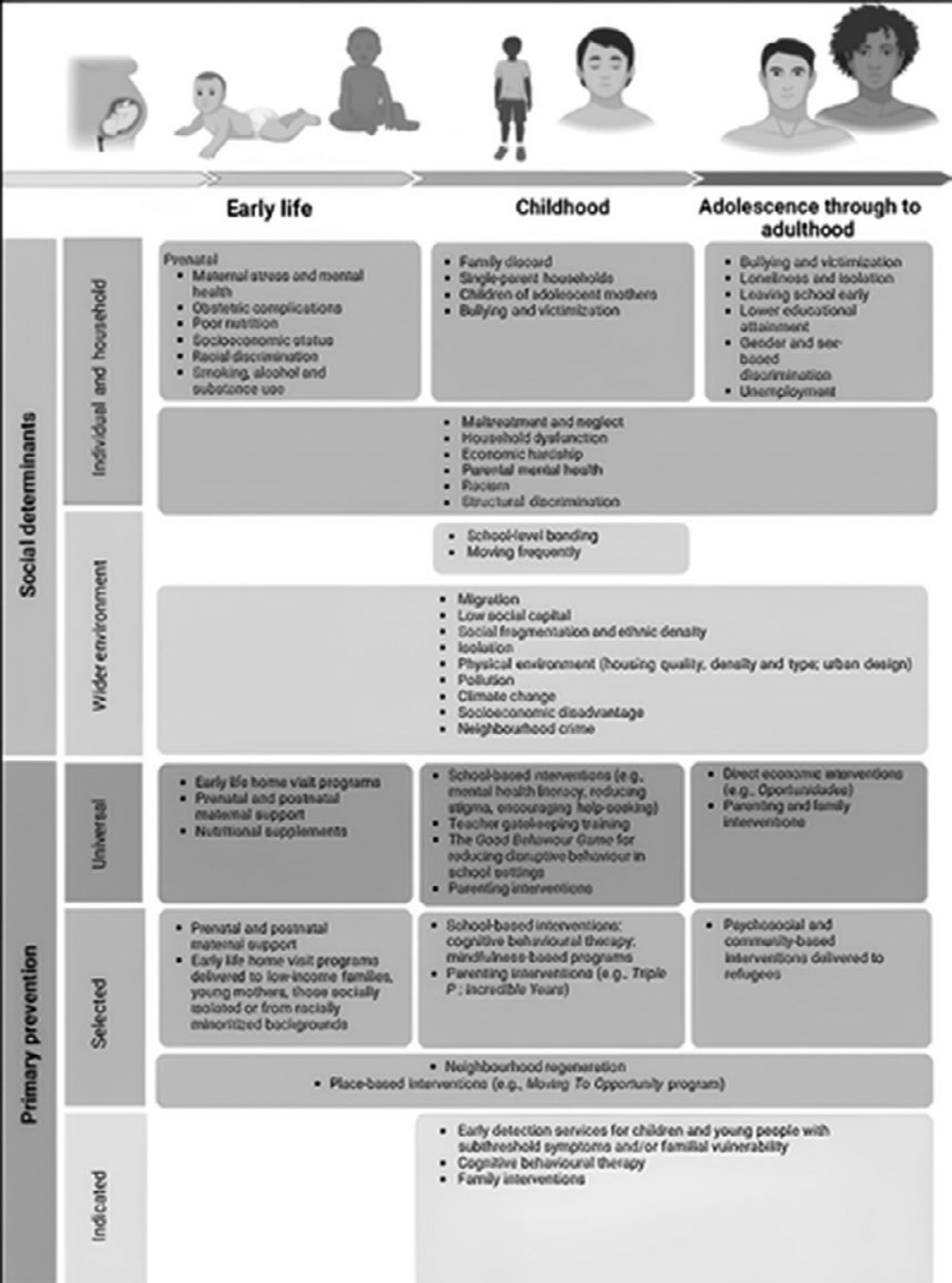
Image 1: Long description.

**Image 2:**
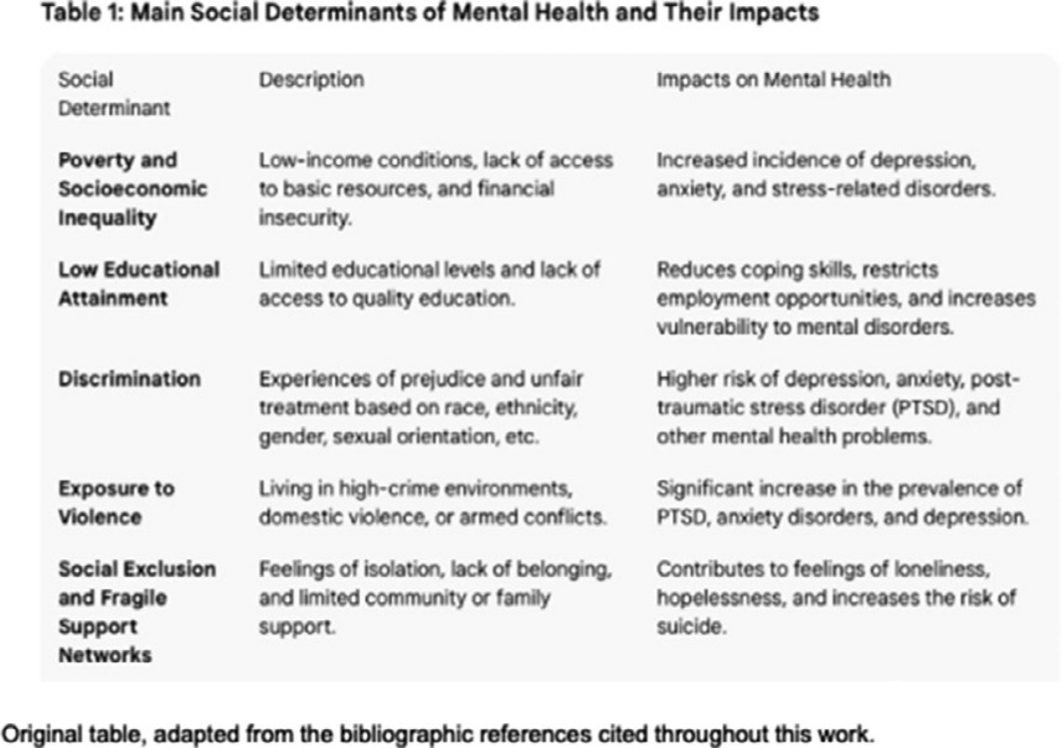
Image 2: Long description.

**Image 3:**
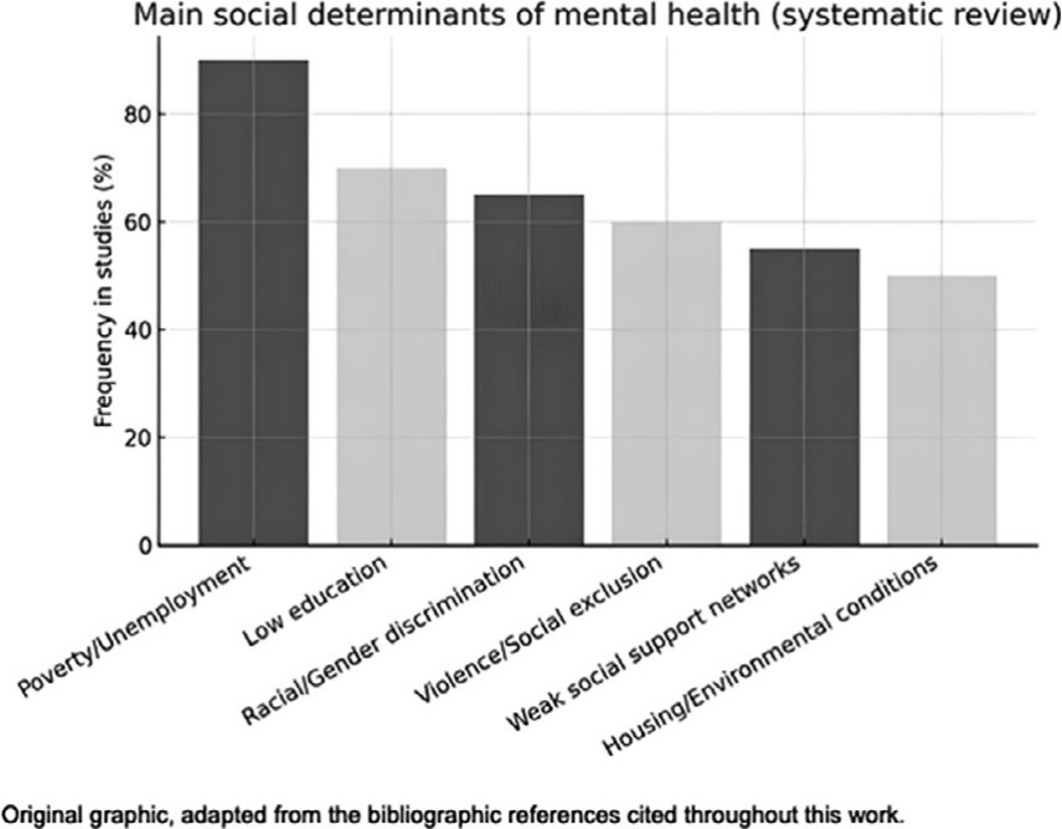
Image 3: Long description.

**Conclusions:**

Thus, factors such as social inequality, discrimination, violence, and precarious living conditions stand out as consistent global predictors, as shown in graphic 1 (World Health Organization and Calouste Gulbenkian Foundation, 2014). Effective mental health interventions must go beyond individual treatment, incorporating public policies that reduce social inequalities, strengthen support networks, and promote social inclusion.

**Disclosure of Interest:**

None Declared

## Long descriptions

### Image 1: Long description

The diagram is organized into a matrix. At the top, a horizontal timeline shows silhouettes of human development from a fetus to adults, divided into three columns: Early life, Childhood, and Adolescence through to adulthood.

Row 1: Social determinants.

- Individual and household factors:

- Early life: Prenatal maternal stress, obstetric complications, poor nutrition, socioeconomic status, racial discrimination, and substance use.

- Childhood: Family discord, single-parent households, children of adolescent mothers, bullying, maltreatment, household dysfunction, economic hardship, parental mental health, racism, and structural discrimination.

- Adolescence through to adulthood: Bullying, loneliness, leaving school early, lower educational attainment, gender and sex-based discrimination, and unemployment.

- Wider environment factors:

- Childhood: School-level bonding, moving frequently, migration, low social capital, social fragmentation, isolation, physical environment, pollution, climate change, socioeconomic disadvantage, and neighborhood crime.

Row 2: Primary prevention.

- Universal interventions:

- Early life: Home visit programs, maternal support, and nutritional supplements.

- Childhood: School-based interventions, teacher gatekeeping training, The Good Behaviour Game, and parenting interventions.

- Adolescence through to adulthood: Direct economic interventions and parenting/family interventions.

- Selected interventions:

- Early life: Prenatal and postnatal maternal support and home visit programs for low-income or racially minoritized backgrounds.

- Childhood: School-based cognitive behavioral therapy and mindfulness programs.

- Adolescence through to adulthood: Psychosocial and community-based interventions for refugees.

- Indicated interventions:

- Childhood: Neighborhood regeneration and place-based interventions.

- Adolescence through to adulthood: Early detection services, cognitive behavioral therapy, and family interventions.

Navigate back to Image 1:.

### Image 2: Long description

The table is organized into three columns: Social Determinant, Description, and Impacts on Mental Health.

* Row 1: Poverty and Socioeconomic Inequality. Description: Low-income conditions, lack of access to basic resources, and financial insecurity. Impacts: Increased incidence of depression, anxiety, and stress-related disorders.

* Row 2: Low Educational Attainment. Description: Limited educational levels and lack of access to quality education. Impacts: Reduces coping skills, restricts employment opportunities, and increases vulnerability to mental disorders.

* Row 3: Discrimination. Description: Experiences of prejudice and unfair treatment based on race, ethnicity, gender, sexual orientation, etc. Impacts: Higher risk of depression, anxiety, post-traumatic stress disorder P T S D, and other mental health problems.

* Row 4: Exposure to Violence. Description: Living in high-crime environments, domestic violence, or armed conflicts. Impacts: Significant increase in the prevalence of P T S D, anxiety disorders, and depression.

* Row 5: Social Exclusion and Fragile Support Networks. Description: Feelings of isolation, lack of belonging, and limited community or family support. Impacts: Contributes to feelings of loneliness, hopelessness, and increases the risk of suicide.

A footer note states: Original table, adapted from the bibliographic references cited throughout this work.

Navigate back to Image 2:.

### Image 3: Long description

The Y-axis is labeled Frequency in studies % and ranges from 0 to 80 in increments of 20, with a final grid line at 90. The X-axis lists six social determinants. The bars alternate between dark gray and light gray, showing a linear decrease in frequency from left to right.

* Poverty/Unemployment: approximately 90%

* Low education: approximately 70%

* Racial/Gender discrimination: approximately 65%

* Violence/Social exclusion: approximately 60%

* Weak social support networks: approximately 55%

* Housing/Environmental conditions: approximately 50%

The title at the top reads Main social determinants of mental health (systematic review). A note at the bottom states Original graphic, adapted from the bibliographic references cited throughout this work.

Navigate back to Image 3:.

